# Fishing in the Soup – Pathogen Detection in Food Safety Using Metabarcoding and Metagenomic Sequencing

**DOI:** 10.3389/fmicb.2019.01805

**Published:** 2019-08-06

**Authors:** Josephine Grützke, Burkhard Malorny, Jens Andre Hammerl, Anne Busch, Simon H. Tausch, Herbert Tomaso, Carlus Deneke

**Affiliations:** ^1^Department of Biological Safety, German Federal Institute for Risk Assessment, Berlin, Germany; ^2^Institute of Bacterial Infections and Zoonoses, Friedrich-Loeffler-Institut, Jena, Germany

**Keywords:** metagenomics, food safety, mock community, bioinformatics, shotgun, 16S, harmonisation, *Francisella tularensis*

## Abstract

In food safety the detection of food contaminations with pathogenic microorganisms is a race against time and often outpaced by error-prone epidemiological approaches. For evidence-based outbreak investigations fast and reliable techniques and procedures are required to identify the source of infection. Metagenomics has the potential to become a powerful tool in the field of modern food safety, since it allows the detection, identification and characterization of a broad range of pathogens in a single experiment without pre-cultivation within a couple of days. Nevertheless, sample handling, sequencing and data analysis are challenging and can introduce errors and biases into the analysis. In order to evaluate the potential of metagenomics in food safety, we generated a mock community containing DNA of foodborne bacteria. Herewith, we compare the aptitude of the two prevalent approaches – 16S rDNA amplicon sequencing and whole genome shotgun sequencing – for the detection of foodborne bacteria using different parameters during sample preparation, sequencing and data analysis. 16S rDNA sequencing did not only result in high deviations from the expected sample composition on genus and species level, but more importantly lacked the detection of several pathogenic species. While shotgun sequencing is more suitable for species detection, abundance estimation, genome assembly and species characterization, the performance can vary depending on the library preparation kit, which was confirmed for a naturally *Francisella tularensis* contaminated game meat sample. The application of the Nextera XT DNA Library Preparation Kit for shotgun sequencing did not only result in lower reference genome recovery and coverage, but also in distortions of the mock community composition. For data analysis, we propose a publicly available workflow for pathogen detection and characterization and demonstrate its benefits on the usability of metagenomic sequencing in food safety by analyzing an authentic metagenomic sample.

## Introduction

During foodborne outbreaks reliable techniques are required to identify the source of infection as fast as possible to prevent further infections with the causative agent. To date, many outbreaks are solved by epidemiological investigations without microbiological evidence. One example for this is the foodborne outbreak of the Shiga toxin-producing *Escherichia coli* of serotype O104:H4 with over 4000 endemic infections and 53 fatal cases in Germany in 2011 (Buchholz et al., 2011). Due to the life-threatening character of this outbreak, a very fast investigation was in demand. Initially, Spanish cucumbers were linked wrongly to the outbreak which led to a drop in vegetable consumption and export of Spanish vegetables with a high economical damage (Burger, 2012). In order to avoid wrong source attribution, microbiological evidence is important. The identification of pathogenic microorganisms is conducted either by targeted or culture-dependent methods. Targeted screening methods as PCR or ELISA can be directly applied without a cultivation step and are therefore very fast but carry the risk of missing atypical strains that are not covered by the applied method. Additionally, these methods do not resolve the affiliation of the detected pathogen to an ongoing outbreak due to a low resolution on molecular level. Whole genome sequencing (WGS) requires a cultivation step in order to receive an isolate from the patient and the contaminated food that are sequenced by next-generation sequencing (NGS). This method has a high discriminatory power and can therefore be used to decipher between outbreak relevant and -irrelevant strains. Currently, WGS is successfully used for source attribution in retro-perspective investigation of foodborne outbreaks (Underwood et al., 2013; Hoffmann et al., 2016; Kleta et al., 2017). However, the isolation process can be too time-consuming for high-throughput screenings of suspected food and is therefore problematic for real-time analysis. The usage of sequencing-based metagenomics allows the simultaneous identification and typing of the causative agent as well as antimicrobial resistance (AMR) or virulence genes and promises to be a very powerful tool for the surveillance of food and drinking water.

The metagenomics analysis is a multi-sequential process and almost every step contains pitfalls that can lead to distorted, blurred and incomplete results. The proper homogenization and cell lysis before nucleic acid extraction has one major impact on the substance of the results. It has to be ensured that all microorganisms have been made accessible for the cell lysis by homogenization and cell lysis reagents are chosen properly for complete access to the nucleic acids (Bag et al., 2016; Knudsen et al., 2016; Wylezich et al., 2018).

The choice of the sequencing method is another decision that could introduce bias into the metagenomic analysis. Two predominant approaches are currently widely used to study the composition of metagenomics samples: a targeted approach using a genetic marker like the 16S rRNA gene for bacteria (Patel, 2001) and a method for the broad-range detection of all pathogens at the same time using the complete genetic information in the sample (shotgun metagenomics). The 16S rRNA gene is the most widely used marker to characterize bacterial communities. This gene is present in the genome of all bacteria and consists of alternating variable and conserved areas. The conserved regions enable the amplification of the nine variable regions using universal primers. The resulting amplicons are prepared for sequencing in a step called library preparation by adding sequences for immobilization, sequencing primer binding sites and DNA barcodes for sample multiplexing. Many studies rely on the sequencing of only one variable region. Therefore the selection of the variable region can influence the results and has to be chosen with care (Chakravorty et al., 2007; Sun et al., 2013; Barb et al., 2016). Shotgun metagenomics aims to gain all genomic information within a sample. The extracted DNA is fragmented and a library is prepared before sequencing. For both fragmentation and library construction, different protocols exist. Either fragments are generated by enzymatic cleavage or by mechanical shearing (e.g., ultrasonication). Meanwhile, dozens of kits for library preparation exist and it is proposed that the choice of the kit can have an impact on the resulting community composition (Bowers et al., 2015; Jones et al., 2015).

One of the major desired applications of metagenomics in food safety is to identify pathogenic microorganisms present in food samples. The basis for this is taxonomic classification that can either be performed by using short reads or longer DNA sequences obtained by the assembly of the sequencing reads (Breitwieser et al., 2017). Additionally, variant analysis might be desired in order to perform pathogen typing to e.g., source attribute contaminated food samples to foodborne outbreaks. Pathogenic agents might not always be among the most abundant species in the sample. As the detection of microorganisms often relies on the presence of genome fragments in the sample, lowly abundant members with few sequencing reads might be missed in the analysis. Additionally, some of the foodborne bacteria e.g., from the *Bacillus cereus* have highly identical genomes and their pathogenicity is determined by virulence factors that are encoded on additional plasmids. In order to assess the risk of contaminated food with these bacteria, specifically their virulence genes have to be detected in a food sample in combination with genomic evidence for their presence.

In this study, we aimed to evaluate the performance of the two predominant metagenomic approaches for their application in foodborne pathogen detection. With the help of a DNA standard consisting of food-associated pathogens, we analyze the impact of the variable regions and the sequencing platform for 16S rDNA amplicon sequencing and the choice of the library kit for shotgun sequencing on the results. Additionally, the proficiency and potential of the prevalent bioinformatics tools for taxonomic classification for each method was analyzed. Our data revealed a superiority of shotgun metagenomic sequencing over 16S rDNA amplicon sequencing in species identification, abundance estimation, sensitivity as well as specificity. But also for shotgun metagenomics sequencing, the choice of the library kit appears to have high impact on the accuracy of the results. We verified our results obtained from the analysis of the DNA standard by exemplarily analyzing a hare liver sample infected with *Francisella tularensis* subsp. *holarctica*. With our knowledge acquired from this study we developed a workflow for pathogen detection and characterization in food samples using shotgun metagenomics sequencing which could pave the way for the usage of metagenomics in food safety.

## Materials and Methods

### Samples and Bacterial Strains

The bacterial *F. tularensis* subsp. *holarctica* isolate 16T0017 was isolated on cysteine heart agar (CHA, Becton Deckinson, BD Heidelberg, Germany) from a carcass of a hare (*Lepus europaeus*) found in 2016 in Rhineland-Palatinate during routine sampling by Friedrich-Loeffler-Institut (Jena), Germany. The liver of the same animal was provided for metagenomic analysis.

### DNA Extraction and DNA Standard

Genomic DNAs (gDNA) used for the DNA standard from isolates belonging to genera *Streptococcus, Staphylococcus*, *Bacillus, Brucella, Escherichia*, *Shigella*, *Burkholderia*, *Salmonella, Klebsiella, Campylobacter, Listeria, Clostridioides, Clostridium, Yersinia, Vibrio, Ochrobactrum* and *Morganella* (Supplementary Table S1) were isolated from plate agar or liquid culture by either using the PureLink^TM^ Genomic DNA Mini Kit (Invitrogen, Carlsbad, CA, United States) or DNeasy Blood & Tissue Kit (Qiagen, Hilden; Germany) according to the manufacturer’s protocol. The isolation of gDNA from *F. tularensis* subsp. *holarctica* isolate 16T0017 and whole DNA from hare (*L. europaeus*) liver was performed as described before (Busch et al., 2018). All extracted DNA was quantified using the Qubit 2.0 fluorometer (Invitrogen, Carlsbad, CA, United States). The determination of *F. tularensis* genome equivalents from whole liver DNA was performed by qPCR as described before (Tomaso et al., 2007). DNA molarity was calculated based on the DNA quantity and the median average length for each species or if available for the strain specified at NCBI genome database. DNAs were combined in an equimolar mixture, containing the same genome copy number (Supplementary Table S2) or the same 16S gene copy number for the copy number normalized mixture for each isolate.

### 16S rDNA Amplification

The amplification of the variable regions (V) of the 16S rDNA was performed with 27F (Lane, 1991) and 338R (Fierer et al., 2008) for V1-2, Bakt_341F (Herlemann et al., 2011) and 533R (Huse et al., 2008) for V3, 520F and 926R (Claesson et al., 2010; Quince et al., 2011) for V4-5, S-D-Bact-0909-a-S-18 and P699R (Klindworth et al., 2013) for V6-7, 1100F and 1492R (Baker et al., 2003) for V7-9 (Supplementary Table S3). The PCR reaction with *Taq* DNA polymerase (Invitrogen, Carlsbad, CA, United States) was prepared according to the manufacturer’s protocol with 2.5 mM MgCl_2_ and 0.8 μM of each primer pair. The PCR amplification was carried out over 35 cycles (30 s at 95°C, 30 s at 50°C, 3 s at 72°C) with an initial 5 min hot start at 95°C and a final extension step (1 min at 72°C). PCR products were purified with Agencourt AMPure XP (Beckman Coulter, Brea, CA, United States) using 1.6× sample volume and quantified using the Qubit 2.0 fluorometer (Invitrogen, Carlsbad, CA, United States).

### Next-Generation Sequencing

DNA libraries for 16S rDNA amplicons sequencing were prepared with the Ion Xpress^TM^ Plus Fragment Library Kit (Ion Torrent, Gilford, NH, United States) or with the Nextera XT DNA Library Preparation Kit (Nextera XT) (Illumina, San Diego, CA, United States) according to the manufacturer’s instructions. 16S rDNA libraries were sequenced with Ion PGM^TM^ using Ion 316^TM^ Chip v2 (Ion Torrent, Gilford, NH, United States) or on the Illumina MiSeq benchtop sequencer in paired-end mode with 2 × 251 cycles using the MiSeq Reagent v3 600-cycle kit (Illumina, San Diego, CA, United States).

DNA from *F. tularensis* subsp. *holarctica* isolate 16T0017 was likewise prepared with the Nextera XT DNA Library Preparation (Nextera XT) Kit and paired-end sequenced with 2 × 300 cycles on the Illumina MiSeq benchtop sequencer. DNA libraries for shotgun sequencing were prepared from the same DNA standard or from whole DNA from hare with Nextera XT DNA Library Preparation (Nextera XT) Kit, Nextera DNA Flex Library Prep (Nextera DNA Flex) Kit, TruSeq Nano DNA Library Prep (TruSeq Nano) Kit (Illumina, San Diego, CA, United States) and ThruPLEX^®^ DNA seq (ThruPLEX) Kit (Takara Bio Inc., Kusatsu, Shiga, Japan) according to the manufacturer’s instructions and pooled prior to sequencing in paired-end mode with 2 × 151 cycles on the NextSeq 500 sequencing system (Illumina, San Diego, CA, United States). Further details on sequenced samples can be found in Supplementary Table S4. All sequences are publicly available at the European Nucleotide Archive (ENA) under the study accession ERP115955.

### Bioinformatics Analysis

Adapters in fastq files generated with PGM were removed after demultiplexing with Cutadapt (Martin, 2011). Paired end reads generated with MiSeq were merged with Qiime (Caporaso et al., 2010) before trimming. Quality trimming of all reads was performed with fastp (Chen et al., 2018) with a mean quality of 30, allowing trimming from both ends for reads generated with the PGM. For random subsampling, seqtk^1^ was used with trimmed reads. For analysis of combined amplicons the same numbers of preprocessed reads from each amplicon after random subsampling were pooled *in silico*. Pooled and individual 16S rDNA amplicon sequences were either classified using kraken2 with default parameters and provided 16S rDNA databases, Qiime2 (Bolyen et al., 2018) with dada2 pipeline and classify-consensus-blast for taxonomic assignment or Qiime with OTU clustering at 99% similarity with cdhit, sortmerna, uclust_ref, or usearch_ref and taxonomic assignment with uclust. The used databases for the 16S rDNA analysis with Qiime and Qiime2 are Greengenes 13.8, Silva v132 and NCBI as downloaded in March 27, 2017. The taxonomic profile from shotgun sequencing was generated with trimmed reads by using MetaPhlAn2 (Truong et al., 2015), kraken (Wood and Salzberg, 2014) and kraken2 with RefSeq v87. For the calculation of the Bray–Curtis dissimilarity indices and assigned reads, abundances of the expected genera and species were extracted. Metagenomic assemblies were generated using MEGAHIT (Li et al., 2015) and reference genome coverage of the resulting contigs was calculated with QUAST (Gurevich et al., 2013). Bray–Curtis dissimilarities were calculated in R with the vegan package (Dixon, 2003). Mapping of all reads to *F. tularensis* subsp. *holarctica* FTNF002-00 (GCF_000017785.1_ASM1778v1_genomic.fna) or to the mock community reference genomes was performed using BWA mem (Li, 2013) and bowtie2 (Langmead and Salzberg, 2012), respectively. Covered bases were extracted with samtools (Li et al., 2009) and bedtools (Quinlan and Hall, 2010). Species specific reads were extracted from kraken2 output by using bbmap (Bushnell, 2014) after the translation of taxids to the full taxonomic lineage with taxonkit (Shen and Xiong, 2019). Kraken2 mpa-reports were filtered for pathogenic species using ABSA database as downloaded in May 2, 2018. From the resulting list, intra-run contaminations were eliminated manually. Taxonomic classification of extracted reads was verified with BLASTn with max_target_seqs 500 and max_hsps 500 parameters in combination with NCBI nt database. For subspecies estimation Mash screen (Ondov et al., 2016) with winner-take-all strategy was run. Virulence factors were identified in species specific reads by using SRST2 (Inouye et al., 2014) with default parameters for strict criteria and a minimum coverage of 60 and minimum depth for the relaxed criteria in combination with the Virulence Factor database (VFDB) (Chen et al., 2005) as downloaded in April 24, 2018. Plots were generated in R with ggplot2, ggtree, cowplot or in MS Excel. The Venn diagram was generated with Venny (Oliveros, 2007) and the workflow diagram was drawn by using Draw.io^2^.

## Results

### Mock Community

**FIGURE 1:**
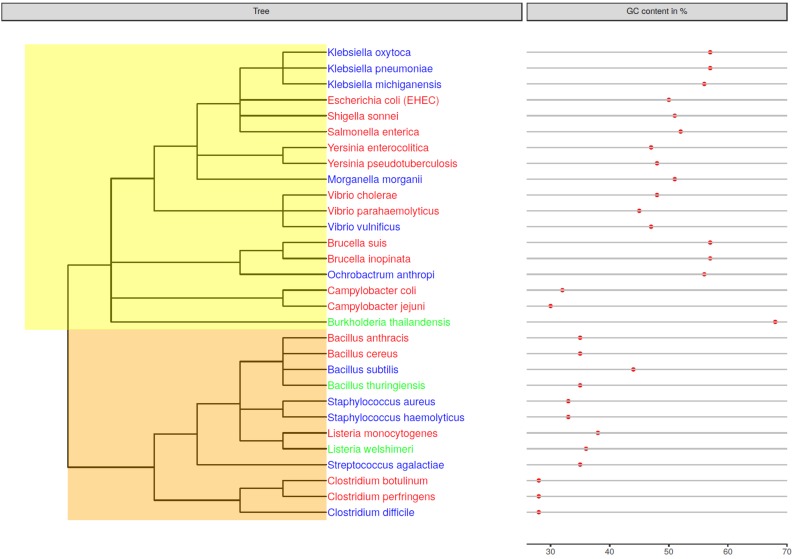
Genetic relatedness of mock community members depicted in a phylogenetic tree. The GC-content of the bacterial genomes is displayed next to the species name. Species names are colored in red for obligate pathogens, in blue for opportunistic pathogens and in green for non-pathogenic species. Nodes were highlighted for gram-negative Proteobacteria (yellow) and gram-positive Firmicutes (orange).

In order to evaluate the usage of metagenomics sequencing methods and analysis tools for the detection of pathogens in food samples a DNA standard was constructed. The standard consists of 34 equimolarly pooled bacterial DNAs belonging to 17 genera and 30 species (Figure 1 and Supplementary Tables S1, S2). The bacterial species were selected based on their incidence in foodstuff and pathogenicity (Tauxe, 2002). In addition to obligate human pathogenic bacteria that cause foodborne illness when ingested, closely related opportunistic or nonpathogenic bacteria were chosen in order to analyze the ability to dissect pathogenic and nonpathogenic species from one another during the analysis (Figure 1). The standard includes 15 strains belonging to the phylum of gram-negative *Firmicutes* and 19 to gram-positive (*Alpha-, Beta-, Gamma- or Epsilon-*) *Proteobacteria*. Except for *B. cereus*, *Clostridium perfringens, Salmonella enterica*, and *Staphylococcus aureus* whereof DNA from two strains were included, all other species are represented by a single isolate. The resulting DNA standard has an average GC-content of 43% and the included genomic DNA ranges between 28 and 68% GC-content (Figure 1).

### 16S rDNA Amplicon Sequencing of a Mock Community

**FIGURE 2:**
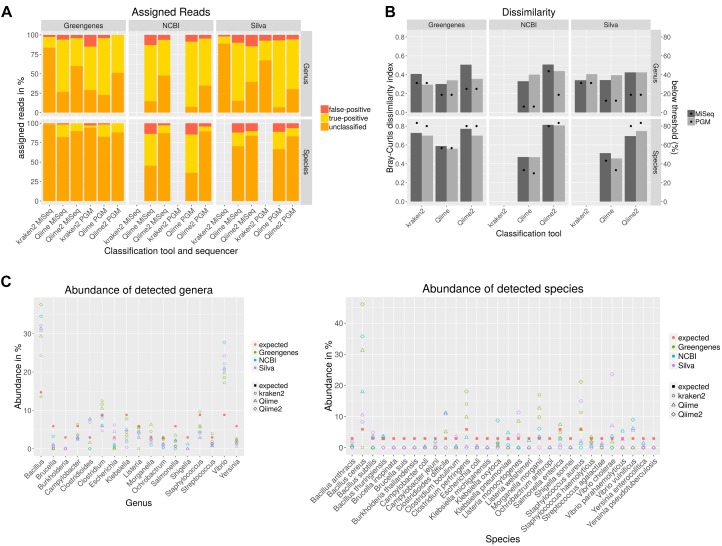
Taxonomic classification of data generated by sequencing all variable regions of the 16S rDNA gene using a mock community standard with indicated databases and classification tools on genus and species level. Unclassified, correctly assigned and false-positive assigned reads are depicted in a staggered bar chart **(A)**. The Bray–Curtis index was calculated after taxonomic classification and represents the dissimilarity to the expected community composition (bars). Dots indicate the percent of mock community members that could be detected only below a threshold of 10% of the expected abundance **(B)**. The abundance for each mock community member from data generated with PGM was calculated **(C)**.

Sequencing of the 16S rDNA is often used to taxonomically- dissect metagenomic samples. Different databases, sequencing technologies and analysis tools were compared by sequencing a mock community to assess the performance of the 16S rDNA amplicon sequencing. For the amplification of the 16S rDNA, published PCR primers with a high overall coverage for bacteria were selected based on a study where 16S rDNA primers were analyzed *in silico* (Klindworth et al., 2013). Taxonomic classification with Qiime was performed using cd-hit for clustering of the operational taxonomic unit (OTU) that shows best results compared to uclust_ref, usearch_ref and sortmerna (Supplementary Figure S1). Bray–Curtis dissimilarities were calculated for combined amplicons (Figure 2B). Higher values indicate higher deviation from the expected composition of the mock community. The dissimilarity index varies less on genus level between used classification tools and databases in the range of 0.3 to 0.5 whereas at species level the dissimilarity index varies highly between 0.46 with Qiime and NCBI database to 0.81 with Qiime2 and NCBI database. NCBI and Silva databases are not available for kraken2 classification or do not include species taxonomy, respectively. For all classification tools and databases some species were only detected at very low abundance (Figure 2C). A detection threshold is set to 10% of the expected abundance for each member of the mock community. Between 6–31% of the genera and 30–83% of species were detected below the detection threshold. Most genera and species above this threshold could be detected with Qiime and NCBI database. Very poor sensitivity especially on species level was obtained after kraken2 and Qiime2 analysis. Some genera were not detected at all (Figure 2C). In particular *Burkholderia* detection with all databases and tools is missing and *Brucella* remained either undetected with Greengenes or is detected at very low abundance. In contrast, the abundance of some genera is highly overestimated as for *Vibrio* and *Bacillus*. On species level, six species remained completely undetected while the abundance of *B. cereus* was highly overestimated. In order to test if the observed overestimation is due to the copy number variation of the 16S gene, a mock community that was normalized to the copy numbers for each species was sequenced with PGM and analyzed with Qiime as described previously. Unsurprisingly, this normalization to the estimated gene copy number obtained from The Ribosomal RNA Database (rrnDB) (Stoddard et al., 2015), decreased the level of over-or underestimation for almost all genera and 14 species and reduced the Bray–Curtis particularly on genus level from 0.4 to 0.26, but reduced the number of undetected only for one species (Supplementary Figure S2). The amount of false-positive assigned reads (Figure 2A) ranges between 0.1 and 15% on genus level and 0 and 14% on species level. When analyzing data with Qiime PGM sequencing resulted in most cases with all databases in a lower false-positive rate than MiSeq sequenced amplicons. The opposite can be observed with kraken2 where MiSeq sequencing showed better results. Using the Greengenes database with all tools resulted in the lowest false-positive rate. In general analysis with Qiime2 resulted in the highest number of unassigned reads and the usage of the Greengenes database produced the highest amount of unclassified reads especially on species level which reflects the previous observation of a higher number of species detected below a threshold of 10% of the expected abundance. The lowest amount of unclassified reads and the highest number of true-positive assigned reads, but also a higher false-positive rate result from the analysis of the data with Qiime and the NCBI database on genus and species level.

In conclusion, no tool in combination with any database was able to detect all members of the mock community. While the sequencing platform did not have a high impact on the results, the selection of the database improved the results as the usage of the NCBI database reduced the amount of false-negative results and showed a lower dissimilarity index but also resulted in a higher false-positive rate.

### Individual 16S rDNA Amplicon Sequencing of a Mock Community

**FIGURE 3:**
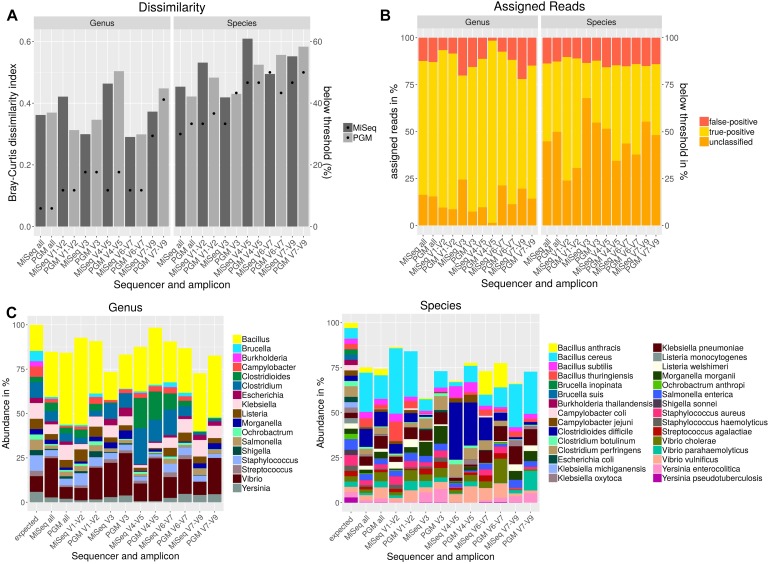
Taxonomic composition after NGS of a mock community standard with PGM and MiSeq using individual amplicons encompassing up to three variable regions (V) of the 16S rDNA gene. Bray–Curtis dissimilarity indices (bars) and the amount of species detected below a threshold of 10% of the expected abundance (dots) are plotted **(A)**. The amounts of false-positive, true-positive and unclassified reads on genus and species level are shown in a staggered bar chart **(B)**. Taxonomic abundance profiling on genus and species level was performed with Qiime and NCBI database for indicated variable regions **(C)**.

Usually, sequencing of only individual amplicons is used for taxonomic classification of metagenomic samples. However, no hypervariable region can be used to differentiate between all bacteria (Chakravorty et al., 2007). In order to determine the most discriminative region for the bacteria included in the mock community individual amplicons sequenced either with PGM or MiSeq were analyzed with Qiime using the NCBI database and cdhit for OTU clustering. Bray–Curtis dissimilarity indices were calculated for OTU tables (Figure 3A) resulting from all individual and from a combination of all amplicons in comparison to the expected abundances of the mock community members. On genus level, lowest dissimilarity indices were obtained when using V3 and V6-V7 region. For most variable regions on genus level, MiSeq sequenced amplicons performed better except for V1-V2 region. The best performance on species level was achieved with V3 region or when all amplicons were used together for the analysis. On species level, better results were obtained with the PGM for variable regions V1-2, V4-5 or when all amplicons were combined and with MiSeq for V3, V6-7 and V7-9. A detection threshold of 10% of the expected abundances was set for all members of the mock community. With this threshold, the most genera and species could be detected when all amplicons were used. Least number of detected genera and species could be observed when V7-V9 was sequenced. For almost all variable regions, *Burkholderia* could only be detected at very low abundances. For *Brucella* spp. and *Ochrobactrum* sp. detection V3 und V7-9 regions performed insufficiently. On species level *Brucella inopinata*, *Brucella suis*, *Klebsiella michiganensis*, and *Shigella sonnei* were not detected at all with any amplicon (Figure 3C). On genus level, high-false positive rates could be observed for regions V3 and V7-V9, while least false-positive assigned reads were obtained for region V1-V2 and V6-7 (Figure 3B). On species level, the amount of reads assigned false-positively is very similar between each amplicon and between the used sequencers with a slightly lower false-positive rate for variable regions V1-V2, V3 or when all amplicons are combined. However, for V3 a large number of reads could not be assigned.

In summary, a combination of all variable regions resulted in the lowest rate of genera and species below the detection threshold and in a higher similarity to the expected mock community composition especially on species level than most of the individually used amplicons. While for V1-V2 a very low false-positive rate and a lower amount of genera and species below the detection threshold was observed, analysis of V7-V9 resulted in a high false-positive rate, the lowest number of genera above the detection threshold and the highest Bray–Curtis dissimilarity to the expected composition of the mock community.

### Shotgun Metagenomic Sequencing of a Mock Community

**FIGURE 4:**
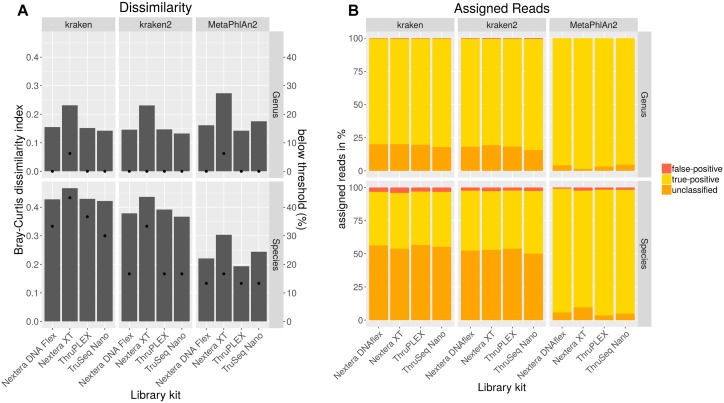
Taxonomic profiling with indicated tools for data generated by shotgun sequencing of a mock community standard processed with indicated DNA library kits. Bray–Curtis dissimilarity indices (bars) were calculated from taxonomic abundance profiles and the amount of genera and species detected below a threshold of 10% of the expected abundance (dots) are shown **(A)**. Proportions of false-positive, correctly assigned and unclassified reads are depicted **(B)**.

In order to analyze if different library kits have an impact on the detection of certain bacteria and community profiling in metagenomics samples by shotgun sequencing, the mock community was sequenced after library preparation with different kits with the NextSeq. The resulting data was quality trimmed and random subsampled to 54 mio reads for each library kit to account for variations in sequencing depth. The data analysis was performed with kraken, kraken2 and MetaPhlAn2 to look for differences between the results outputted by these frequently used taxonomic classification tools. Bray–Curtis dissimilarity indices were calculated for resulting taxonomic abundance tables in comparison to the expected abundances for the mock community (Figure 4A). On genus level, the dissimilarity index ranges between 0.13 and 0.27 with lowest values for the TruSeq Nano library kit in combination with kraken2. With all analysis tools for both genus and species level highest dissimilarities were obtained with the Nextera XT kit, while lowest dissimilarity indices were calculated when the TruSeq Nano kit was used except for data analyzed with MetaPhlAn2 where lower dissimilarity was achieved with the ThruPLEX library kit. On species level, the dissimilarity indices range between 0.19 and 0.47 over library kits and taxonomic classification tools. Here, lowest dissimilarities were calculated after analysis with MetaPhlAn2. All genera included in the mock community could be detected with all tools however, *Clostridioides* was not detected above a detection threshold of 10% of the expected abundance when the Nextera XT kit was used (Figure 4A). Least species below the detection threshold (13–17%) were detected when the data was analyzed with MetaPhlAn2. With kraken2 17–33% and kraken 33–46% of the species were not detected above the detection threshold. Generally 1–5 species less above the detection threshold were detected when the Nextera XT kit was used as compared to the other kits. Between 0 and 0.5% of the genera were assigned false-positive (Figure 4B). No genus was assigned wrongly with MetaPhlAn2. On species level the false-positive rate is between 0.9–2.4 for MetaPhlAn2, 2.6–2.8 for kraken2, and 3.1–4.1 for kraken. The amount of unassigned reads between kraken and kraken2 on genus and species level is very similar between 50 and 57%. A very low rate of unassigned species and genus can be detected for MetaPhlAn2. However, a read-wise assignment is not feasible with MetaPhlAn2 where abundance estimation is realized by unique genomic marker detection (Truong et al., 2015).

**FIGURE 5:**
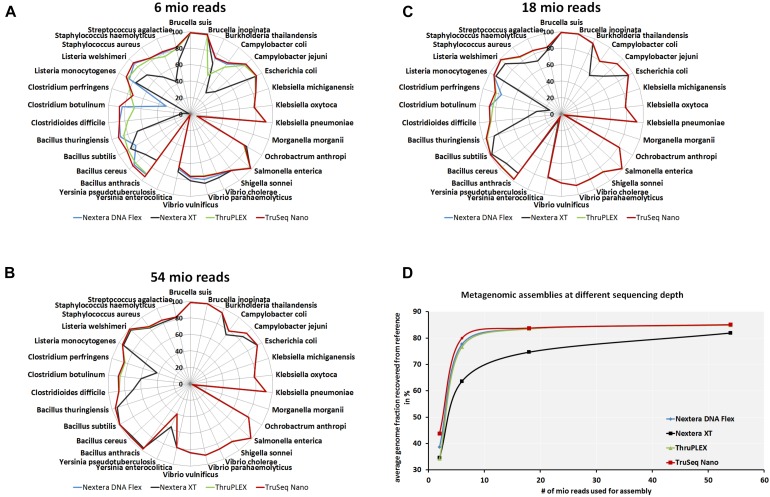
Completeness of metagenomic assemblies depending on sequencing depth and library kits. Assemblies were generated after shotgun sequencing of a mock community with indicated DNA library kits with 6 × 10^6^**(A)**, 1.8 × 10^7^ **(B)** and 5.4 × 10^7^**(C)** reads. Relative completeness of the assembled genomes in comparison to the reference genomes in percent are plotted in spider diagrams. The recovered genome fractions from reference genomes for all species included in the mock community where averaged for at indicated read depths **(D)**.

To analyze if the usage of different library kits has consequences on the completeness of draft genome assemblies from the mock community members, metagenomics assemblies were generated at different read depths. The recovered genome fraction calculated by comparing the assembled draft genomes and reference genomes for each species in the mock community (Figures 5A–C) show few differences between library kits for some species as *S. enterica*, *Ochrobactrum anthropi*, *Klebsiella* spp., *E. coli*, *Brucella* spp. and *Vibrio* spp. Larger differences can be observed even at very high read depth of 54 million reads (Figure 5A) for *Bacillus anthracis*, *B. cereus, Bacillus subtillis*, *C. perfringens*, *Clostridium* spp. and *Campylobacter* spp. with lower amount of recovered genome fraction when the Nextera XT kit was used. At lower read depth (6 and 18 million reads) even more species belonging to the genera *Listeria*, *Staphylococcus* and *Streptococcus* have a lower genome recovery after the assembly with the Nextera XT kit as compared to the other three library kits. When comparing the total recovered genomes from all species of the mock community (Figure 5C) at different read depths a superiority of the TruSeq Nano Kit closely followed by the Nextera Flex and the TruPLEX kits over the Nextera XT kit can be observed.

**FIGURE 6:**
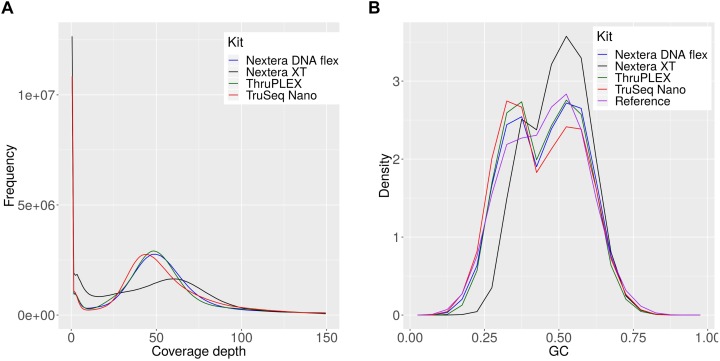
Coverage depth and GC density histograms for reads obtained by sequencing a mock community after library preparation with indicated library kits. The depth histograms were obtained by read-mapping of the shotgun data to the set of mock-community reference **(A)**. The GC-compositions are plotted as density histograms for reads and 150 bp long genome fragments for the reference genomes **(B)**.

The observed differences for the Nextera XT kit were further investigated and 54 million reads per sample were mapped against the references of all members of the mock community (Supplementary Table S1) for each library kit. The comparison of the coverage depths of the references genomes between the kits revealed a high number of uncovered regions for the Nextera XT kit and a higher dispersion of coverage depth (Figure 6A), which indicates a favored generation of fragments for certain genome regions, that occurs during library preparation (Figure 6A). Therefore the GC content of the generated reads was compared between the library kits (Figure 6B). With this we were able to detect a shift in the GC density of fragments generated with the Nextera XT kit to higher GC-contents, while this was balanced for the other three library kits and in accordance with the expected distribution from the collection of mock-community reference genomes. To sum up, the choice of the library kit can strongly influence the performance of shotgun metagenomics sequencing. The Nextera XT kit performed less efficiently for species detection and abundance profiling and for many species the genome can be recovered much better with the TruSeq Nano, TruPLEX and the Nextera Flex kit, which can be explained by the GC-bias of the Nextera XT kit. MetaPhlAn2 performed better than kraken2 and kraken for the taxomomic profiling – however, the unique genomic marker database that is the basis for this tool cannot easily be expanded.

### Comparison of 16S rDNA Amplicon Sequencing and Shotgun Sequencing of a Mock Community

**FIGURE 7:**
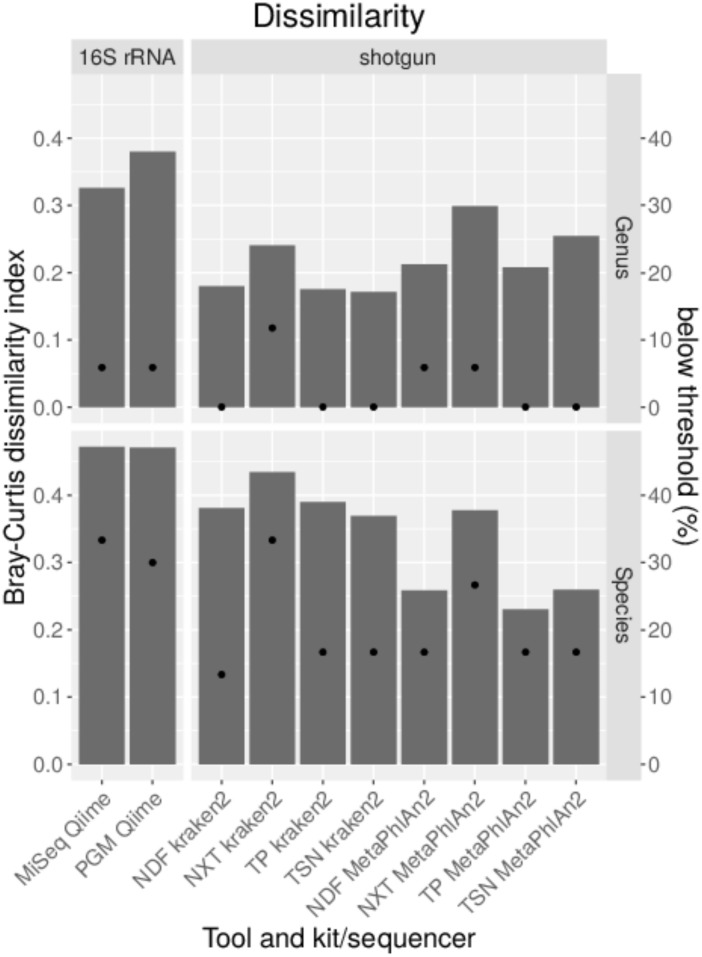
Comparison of the taxonomic profile generated with data from shotgun sequencing with indicated DNA library kits and 16S rDNA sequencing with indicated sequencing platforms of a mock community standard. 100,000 reads were subsampled from data of both applications. Bray–Curtis dissimilarity indices (bars) were calculated from taxonomic abundance profiles and the amount of genera and species detected below a threshold of 10% of the expected abundance (dots) are shown.

For the comparison of shotgun and 16S rDNA amplicon sequencing, 100,000 reads were classified for each method with parameters that performed well previously. Bray–Curtis dissimilarities were calculated (Figure 5). By comparing results obtained from shotgun metagenomics sequencing to 16S rDNA amplicon sequencing, much smaller Bray–Curtis dissimilarity indices can be obtained with shotgun sequencing on genus and species level except when the DNA-library was prepared with the Nextera XT library kit that results in much higher dissimilarity indices. When excluding the Nextera XT library kit, the average Bray–Curtis dissimilarity on genus level is 0.23 with MetaPhlAn2 and 0.18 with kraken2 as compared to a much higher dissimilarity of 0.35 for 16S rDNA amplicon sequencing of the mock community. On species level, a smaller average Bray–Curtis index is observed when MetaPhlAn2 was used (0.28), compared to kraken2 (0.39). The largest dissimilarity was calculated for the 16S rDNA amplicon sequencing with a Bray–Curtis index of 0.47 independent from the sequencing platform (Figure 7). A detection threshold was set to 10% of the expected abundance. All genera could be detected when shotgun libraries were either prepared with the TruSeq Nano or the ThruPLEX kit. While for shotgun sequencing *Clostridium* could not be detected above the detection threshold with the Nextera XT and Nextera DNA Flex kit, *Brucella* was below the detection threshold for 16S rDNA amplicon sequencing. On species level > 30% of the species are not detected with 16S rDNA amplicon sequencing in comparison to the shotgun sequencing where in average 17% species are below detection threshold when Nextera XT kit is excluded. No method was able to detect *B. suis* and *Yersinia pseudotuberculosis*. With kraken2 *O. anthropi* and *Bacillus thuringiensis* were detected at very low abundance, while MetaPhlAn2 failed to detect *K. michiganensis* and *Clostridium botulinum* completely. With 16S rDNA amplicon sequencing and the analysis with Qiime additionally *B. inopinata*, *Burkholderia thailandensis*, *E. coli*, *K. michiganensis*, *S. sonnei* as well as *Listeria welshimeri* were not detected above the detection threshold.

Shotgun sequencing provides much lower dissimilarity indices on average, especially on species level as obtained after 16S rDNA amplicon sequencing. More importantly, more genera and species above the detection threshold could be detected with shotgun sequencing. However, the results obtained with the Nextera XT kit are less reliable in accordance to the previously described results.

### Detection of a Highly Pathogenic Bacterium, *F. tularensis* Subsp. *holarctica* in Game Meat Using Metagenomics Shotgun Sequencing

**FIGURE 8:**
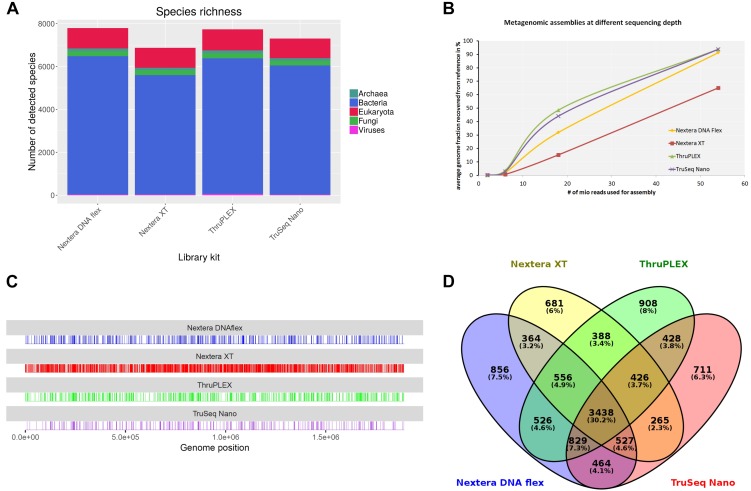
Analysis of a hare liver sample infected with *Francisella tularensis* after shotgun sequencing with indicated DNA-library kits after sampling 5.4 × 10^7^ reads for each kit. Species richness for different domains of life was determined **(A)**. From metagenomics assemblies the completeness of the *F. tularensis* subsp. *holarctica* genome compared to the reference genome was calculated at different sequencing depths **(B)**. Reads were mapped to *F. tularensis* reference genome and unmapped bases along the genome are shown as vertical lines **(C)**. Venn diagram shows overlap between species detected with kraken2 from data generated with different DNA library kits **(D)**.

For the verification of the previously obtained results originating from the shotgun sequencing of a mock community, an authentic metagenomic sample was sequenced. The sample was collected from a wild hare liver in Germany infected with *F. tularensis* subsp. *holarctica*. In parallel to DNA extraction, a *F. tularensis* subsp. *holarctica* isolate was recovered from this sample. *F. tularensis* subsp. *holarctica* was detected via real-time PCR targeting the *tul4* gene that is specific to *F. tularensis* as described previously (Versage et al., 2003). Via extrapolation of a standard curve, 1.2 × 10^7^ genome equivalents were calculated. DNA libraries were prepared using the Nextera DNA Flex, Nextera XT, ThruPLEX and TruSeq Nano kits. At different read depths obtained by subsampling the original data to 6 million, 18 million and 54 million reads, library kits and analysis tools were tested. Of all tested tools, only kraken2 that can be used with the complete RefSeq database enables a taxonomic classification referring to the whole metagenomic sample. Independent of the read depths *F. tularensis* was detected with a relative abundance between 0.1 and 0.3%. The lowest amount of *F. tularensis* assigned reads (0.1%) was obtained when the Nextera XT kit was used and highest amount of reads (0.3%) when the ThruPLEX library kit was used. 99% of the reads were assigned to *Mammalia* whereof 97% were assigned to *Oryctolagus cuniculus*. MetaPhlAn2 was similarly able to identify *F. tularensis* in the sample. Here the abundance of *F. tularensis* was between 43% with the TruSeq Nano kit and 63% with the Nextera DNA Flex kit. Although MetaPhlAn2 is able to identify strains, no strain or subspecies was detected with all kits. The highest amount of bacteria species was detected with kraken2 when the library was prepared with the Nextera DNA Flex kit (6,445 species) while least species were detected with the Nextera XT Kit (5,561 species) (Figure 8A). In the other domains of life, the number of detected species was very similar between the library kits. It is worth mentioning that the highest number of detected viral species could be obtained with the ThruPLEX kit which correlates with the overall number of classified viral reads. Only 3,438 bacteria species (< 62%) are shared after classification with kraken2 when all four library kits are compared (Figure 8D).

Metagenomic assembly at different read depths was performed in order to analyze how the performance of the different library kits influences the completeness of the *F. tularensis* draft genome (Figure 8B). All assemblies were compared to the complete genome of *F. tularensis* subsp. *holarctica* FTNF002-00 (Barabote et al., 2009) that was determined as the most similar reference genome to the isolate obtained from the metagenomic sample with Mash. With 2 and 6 million sequenced reads, only a very small proportion of the reference genome could be detected. At the highest tested read depth of 54 million reads, the assemblies from the ThruPLEX and TruSeq Nano kit were similarly able to cover 94% of the reference genome. Also with the Nextera DNA Flex kit, > 90% of the reference genome could be recovered. With the Nextera XT kit however, only 65% of the reference genome is assembled. Reads classified as *F. tularensis* with kraken2 were extracted and mapped to the *F. tularensis* subsp. *holarctica* FTNF002-00 complete genome (Figure 8C). The percentage of uncovered bases was 1.4% with the TruSeq Nano kit and similar between the ThruPLEX and the Nextera DNA Flex kit with 2.3–2.9% while with the Nextera XT kit around 12% of the bases were not covered.

In summary, it was possible to detect *F. tularensis* in an authentic metagenomic sample with all library kits. The lowest proportion of *F. tularensis* reads was detected with the Nextera XT kit when classification was performed with kraken2. The highest species richness could be similarly obtained with the Nextera DNA Flex and the ThruPLEX kit. When comparing the recovery of the *F. tularensis* reference genome after performing metagenomic read assembly the higher genome fraction could be obtained when the TruSeq Nano and the ThruPLEX kit were used, while even at high read depth 30% less of the reference genome could be recovered when the Nextera XT kit was used. Corresponding results were obtained when reads were mapped to the reference genome.

### Workflow for the Detection and Characterization of Pathogenic Bacteria by Metagenomic Shotgun Sequencing

**FIGURE 9:**
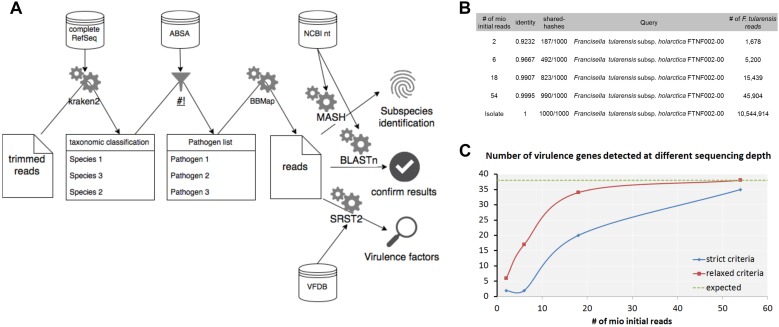
Workflow for the detection and characterization of pathogenic microorganisms using shotgun metagenomics sequencing. Taxonomic profiles are generated with kraken2 using the complete RefSeq database. The resulting taxonomic abundance table is filtered for pathogenic species using ABSA database. For detected pathogens classified reads are extracted and verified with BLASTn using the nt database from NCBI. Subspecies is resolved by determination of closest available reference using Mash. Virulence factors are detected with SRST2 in combination with the VFDB. **(A)** Analysis of a hare liver sample infected with *F. tularensis* subsp. *holarctica* using the workflow shows determination of the *F. tularensis* subspecies **(B)** and the detection of virulence factors **(C)** at different read depths from metagenomic sequencing data and WGS data of the isolate extracted from the same hare liver sample.

Detection of pathogenic bacteria in metagenomic samples usually comprises a step of taxonomic profiling of shotgun sequencing data with bioinformatics tools. As shown in this study, taxonomic profiling might result in false-positive and false-negative assignments of sequences especially on species level. Therefore, less pathogenic or opportunistic species could be detected instead of human pathogenic species that would result in an underestimation of the risk potential and *vice versa*. A verification of the classification results is hence necessary. Additionally, the species level that is the output of most classifiers might not be sufficient to assess the risk of foodstuffs; therefore it is indispensable to determine resistance and virulence genes from the dataset as well as the closest reference genomes for potential pathogens. Another problem of the taxonomic classification is the complexity of the outputted species lists, which comprise between hundreds and thousands of species for metagenomic samples. In order to find species relevant for risk assessment, it is necessary to filter for pathogenic microorganisms automatically which will remove irrelevant information for risk assessment and help to reduce the complexity of the analysis. Here we propose a metagenomic analysis workflow for the microbial risk assessment of food samples (Figure 9A).

First steps include the trimming of the raw data for low quality bases in the reads and classification of trimmed reads with kraken2 using the complete RefSeq. In our experience, smaller databases that include only subsets of eukaryotic genomes increase the false-positive classification rate immensely. The resulting species list is filtered for human, animal or plant pathogens as well as for select CDC or USDA agents using the risk group database from the American Biological Safety Association (ABSA) as it was shown before for viruses in clinical samples (Tausch et al., 2018). For selected pathogens of interest, classified reads are extracted from the metagenomic dataset in order to (i) verify the classification with BLASTn and the nucleotide database from NCBI, (ii) estimate the closest distance to a published reference genome with Mash for subspecies identification and (iii) identify virulence factors using SRST2 using the Virulence Factor Database (VFDB). The proposed workflow is publicly available at gitlab^3^.

For *F. tularensis* subsp. *holarctica* infected hare liver sample sequenced with the four library kits the initial taxonomic classification list with > 7,000 species was reduced to 323–393 species by filtering for human pathogenic species. Only eight human pathogenic species could be confirmed by BLASTn. Thereof only *F. tularensis* and *F. philomiragia* were detected with all four library kits and the opportunistic pathogens *Moraxella osloensis* was detected with two library kits (Table 1). Each further pathogenic species was detected with only one library kit and only one confirmed read.

**TABLE 1:** BLASTn results for detected and ABSA-filtered human pathogens with kraken2 classified reads for each library kit applied for shotgun sequencing of a *F. tularensis* contaminated hare liver: Nextera DNA Flex (NDF), Nextera XT (NXT), ThruPLEX (TP) and TruSeq Nano (TSN).

**Species**	**NDF**	**NXT**	**TP**	**TSN**
*Francisella tularensis*	999/1000	992/1000	960/1000	998/1000
*Francisella philomiragia*	14/38	3/12	26/76	12/40
*Arcobacter butzleri*	1/22	0/0	0/0	0/0
*Streptococcus suis*	1/48	0/0	0/0	0/0
*Moraxella osloensis*	0/0	5/20	2/12	0
*Acanthamoeba castellanii*	1/50	0/0	0/0	0/0
*Atopobium parvulum*	1/2	0/0	0/0	0/0
*Streptococcus gallolyticus*	0/0	0/0	0/0	1/10

*Numbers indicate how many reads could be verified with BLASTn from total number of kraken2 classified input reads.*

Further characterization using Mash for *F. tularensis* provided the closest reference available at NCBI *F. tularensis* subsp. *holarctica* FTNF002-00 already at a total read depth of 2 × 10^6^ and is in concordance for what was found for isolate 16T0017 extracted from the same sample (Figure 9B). Thirty-nine virulence factors (VFs) could be detected with SRST2 and VFDB for the isolate 16T0017 (Figure 9C). With strict criteria applied for WGS analysis of isolated strains only 35 VFs were identified at highest read depths for the metagenomics sample while all VFs were found when coverage and depths were reduced (relaxed criteria).

In summary, our workflow enables the reduction of complex output generated by primary data analysis tools to relevant information for food safety that simplifies the risk assessment of foodstuffs using metagenomics sequencing. We were able to characterize *F. tularensis* to subspecies level already at low sequencing depths; however, the recovery of virulence factors requires higher genome coverage of microorganisms.

## Discussion

In this study a mock community DNA standard was generated and used to broadly evaluate metagenomic sequencing methods for the detection of foodborne pathogens for microbial risk assessment of foodstuffs. Here, the predominant metagenomic sequencing methods 16S rDNA amplicon and shotgun sequencing as well as several parameters that can distort the analysis including variable regions of the 16S rRNA gene, library preparation protocols, sequencing platform, sequencing depths, clustering, taxonomic classification tools and sequence databases were tested. 16S rDNA amplicon data is generated and analyzed in many studies and it can be useful for the detection of pathogenic bacteria in foodstuffs because the dominant eukaryotic DNA originating from the food matrix is excluded. However, this method relies on small nucleotide differences between genera/species within a short region of 200–300 bp in size and is hence susceptible to the introduction of wrong nucleotides by polymerases during the amplification. Another downside is that the 16S rRNA gene is not a single-copy gene in most bacteria and its copy number varies between the species and genera, so that the relative abundance cannot be directly derived from this data (Louca et al., 2018). However, a better abundance profile might be obtained by normalizing the data to the 16S rRNA gene copy number, as we observed it when the copy number normalized mock community members was sequenced, however, well-performing tools to correct the data are still lacking (Louca et al., 2018).

For 16S rDNA amplicon analysis Qiime and its successor Qiime2 as one of the most used tools for this application as well as kraken2 that is the successor of kraken and now offers 16S rDNA databases to classify 16S rDNA amplicon data were chosen for taxonomic classification in this study. At genus level all tools performed similar. The only database for kraken2 that includes the species level is Greengenes. At species level, Qiime performed better than Qiime2 and kraken2 with the Greengenes database. A side-by side comparison of Qiime and Qiime2 was never performed, however, two studies comparing OTU (offered by Qiime) and ASV (offered by Qiime2/dada2) approaches report lower amount of ASVs than OTUs (Allali et al., 2017; Nearing et al., 2018), which might explain missed genera and species that in turn are also the reason for the higher Bray–Curtis dissimilarity values for Qiime2. The Greengenes database is comparatively incomplete and has not been updated since 2013 (Balvociute and Huson, 2017). In our study, many species could not be detected at all with this database. Nevertheless, this database is still provided by recently developed tools as kraken2 and Qiime2.

The similarity to the expected composition and the number of detected species was highest when NCBI database was used. Furthermore the usage of a combination of all variable regions improves the detection of pathogenic bacteria. If only one region can be used e.g., due to limited amount of sample, the V1-V2 region performs better for genus and species detection than other regions. The sequencing platform does not influence the results to a high extend and none is consistently superior. Our study shows that 16S rDNA amplicon sequencing is rather unsuitable for the detection of pathogens especially on species level. On genus level all genera except for *Burkholderia* could be detected. However, for several bacteria, genus level is not sufficient when pathogenicity varies to a substantial extent between species within one genus, e.g., *Listeria monocytogenes* that causes listeriosis after ingestion of contaminated food products and the apathogenic *L. welshimeri*.

Shotgun sequencing resulted in a much better genus and species detection as well as higher similarity to the expected taxonomic composition for the mock community compared to 16S rDNA amplicon sequencing. The detection was very similar between kraken2 and MetaPhlAn2, but the abundance estimation of MetaPhlAn2 was closer to the expected abundance on species level. MetaPhlAn2 provides fewer false-positive results as kraken or kraken2 and is less computation-intensive. However, its own database with clade-specific unique marker genes cannot be extended independently and e.g., for *Brucella* genus human pathogenic *B. suis, B. abortus, B. melitensis, B. canis* and *B. neotomae* species are not included, which leads to false-negative results and misclassification if these *Brucella* species are present in the sample. Admittedly, *Brucella* exhibit a low genetic diversity between species (Scholz and Vergnaud, 2013) and unique genetic markers genes might be difficult to identify. Therefore species assignment within the *Brucella* genus can rather be performed on the level of single nucleotide differences. A promising novel tool, mOTUs2, that uses marker genes in combination with single nucleotide variation profiles and offers an extendable database might be able to cope with this issue (Milanese et al., 2019). However, with these approaches, detection of microorganisms relies on the presence of marker genes which requires these genes to be sequenced and can be problematic for low abundant pathogens. Kraken and kraken2 offer the opportunity to construct custom databases. With kraken2, it is now possible to use even very large genome databases such as the complete RefSeq and thereby provides an opportunity to also identify the matrix signals and to detect pathogens from all domains of life at once. This may still yield false-positive classifications as it can be observed when classified reads for human pathogens are verified with BLASTn. Hence, we recommend verifying the kraken2 results for pathogenic species via BLASTn as we propose with our workflow.

As we could show in this study, the choice of the library kit belongs to one of the key considerations for pathogen detection and characterization in metagenomics food samples when shotgun sequencing is applied. Fewer species above the detection threshold can be observed and the community composition seems to be biased after sequencing with the Nextera XT kit. When further analysis as genome assembly is required, the lack of performance with this kit is even more obvious as it dramatically reduces the genome recovery for some of the bacteria species. The analysis of the genome coverage of the *F. tularensis* subsp. *holarctica* genome with similar amount of input reads show that over 8% of the genome is lacking with the Nextera XT kit compared to the other three library kits. We could show that these observed shortcomings after library preparation with the Nextera XT kit probably stem from a GC bias towards genome regions with higher GC-contents and might result from the transposase insertion bias as it was proposed for HLA genotype calling (Lan et al., 2015). It is however, a new finding that this transposase insertion bias can be apparently completely suppressed in metagenomics samples by the application of bead linked transposases that bind DNA and likely force the cleavage reaction as in the Nextera DNA flex kit (Bruinsma et al., 2018).When comparing the amount of all detected species in the game meat sample, most species are detected with the Nextera DNA Flex and the ThruPLEX, whereas more than 900 species less can be detected with the Nextera XT kit, which affects the domain of prokaryotes in particular. This suggests that the choice of the library kit not only affects the genome coverage, but might lead to an under- or overestimation of species richness in metagenomics studies. Interestingly, mutually- exclusive species ranging from 6 to 8% of all detected species can be observed with all kits.

In order to detect pathogens in foodstuffs by metagenomics we recommend using shotgun sequencing as it is universally- applicable for the detection of microorganisms from all domains of life and allows further characterization of the detected species. The resulting data facilitate resolution to species and subspecies level and can therefore be applied for outbreak investigations. The results produced by taxonomic classification however, need to be reviewed because of false-positive classifications. With our workflow, false-positive results are removed and the complexity of vast species lists is reduced to relevant information for microbial risk assessment. Moreover, the inclusion of pathogen characterization by virulence factor analysis and subspecies estimation opens the door for avoiding long isolation procedure for infectious agents by using metagenomics in food safety.

## Data Availability

The datasets analyzed for this study can be found in the European Nucleotide Archive under the study accession ERP115955 (https://www.ebi.ac.uk/ena/data/view/PRJEB33186).

## Author Contributions

JG, BM, CD, and JH conceived the study. JH and JG designed and constructed the mock community standard. JG performed metagenomics sequencing of the mock community and the hare liver sample. AB performed sequencing and data curation of the *F. tularensis* subsp. *holarctica* isolate and involved in conceptuation. JG, CD, and ST performed bioinformatics analyses and designed the workflow. HT performed sample selection and project administration. JG interpreted the data and wrote the manuscript. All authors were involved in project discussion and approved the final manuscript.

## Conflict of Interest Statement

The authors declare that the research was conducted in the absence of any commercial or financial relationships that could be construed as a potential conflict of interest.

## Footnotes

1 https://github.com/lh3/seqtk

2 https://www.draw.io/

3 https://gitlab.com/bfr_bioinformatics/foodsafetymetagenomics
