# Supplementary material for: Fishing in the Soup – Pathogen Detection in Food Safety Using Metabarcoding and Metagenomic Sequencing
Source: Front Microbiol. 2019 Aug 6;10:1805. doi: 10.3389/fmicb.2019.01805 (PMC6691356; doi:10.3389/fmicb.2019.01805)
Supplement: Supplementary file 1 [file Table_1.docx]

Supplementary Material


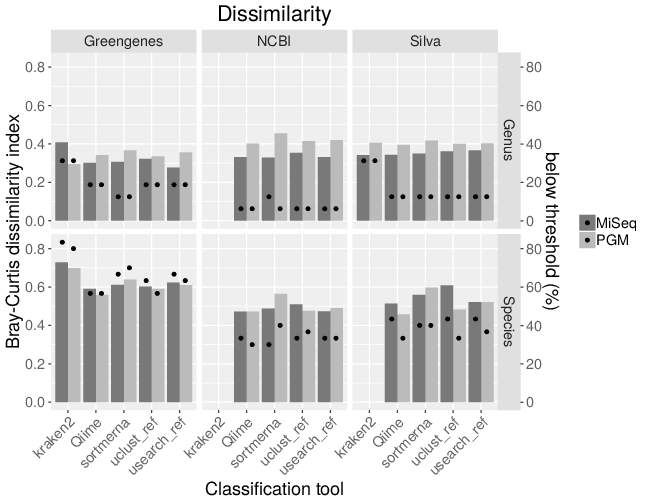


**Supplementary Figure 1.** All variable regions of the 16S rDNA gene using a mock community standard were sequenced and OTU tables were generated with Qiime and kraken2. For indicated databases, sequencers and clustering algorithms the Bray-Curtis dissimilarities were calculated in comparison to the expected abundances of the mock community members (bars). Dots indicate the percent of mock community members that could be detected only below a threshold of 10% of the expected abundance

**
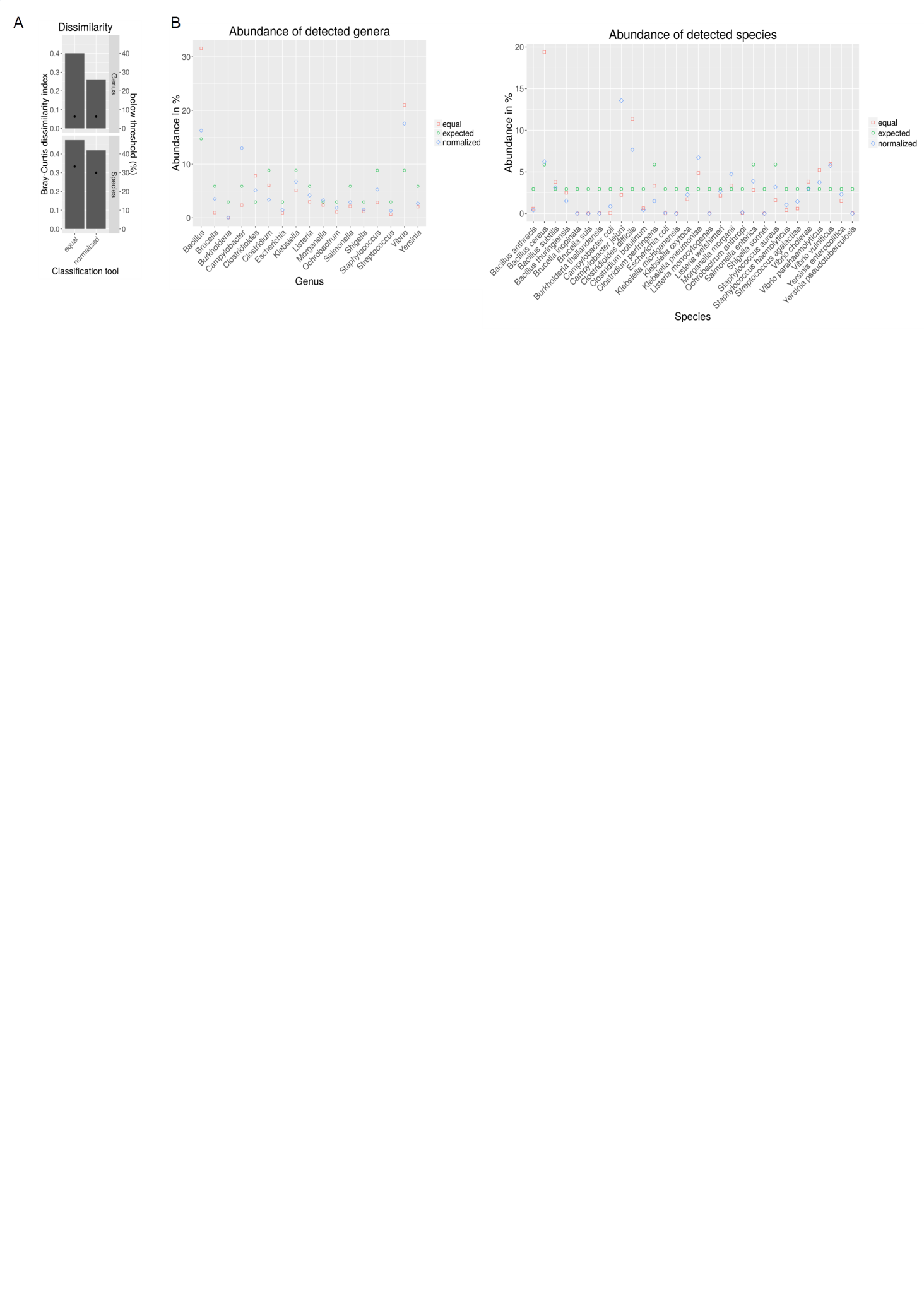
**

**Supplementary Figure 2.**

Taxonomic classification of data generated on PGM by sequencing all variable regions of the 16S rDNA gene using a mock community standard in an equimolar and copy number normalized mixture on genus and species level. Bray-Curtis dissimilarity indices (bars) and the amount of species and genera detected below a threshold of 10% of the expected abundance (dots) are plotted (A). The abundance for each mock community member was calculated using Qiime and NCBI database (B).

**Supplementary Table 1**. Strains included in mock community standard with assembly accession and BioSample ID if available.

**Supplementary Table 2.** Calculated number of genome equivalents in the equimolar pool of mock community members used as input for each kit.

**Supplementary Table 3.** Primer used for 16S rDNA amplicon generation.

**Supplementary Table 4.** Technical specifications for each sample, library preparation and sequencing
